# Cyclists do not need to incorporate off-bike resistance training to increase strength, muscle-tendon structure, and pedaling performance: Exploring a high-intensity on-bike method

**DOI:** 10.5114/biolsport.2025.146790

**Published:** 2025-02-05

**Authors:** Jesús G. Pallares, David Barranco-Gil, Víctor Rodríguez-Rielves, Raúl de Pablos, Ángel Buendía-Romero, Alejandro Martínez-Cava, Francisco Franco-López, Iván R. Sánchez-Redondo, Jon Iriberri, Carlos Revuelta, José Ramón Lillo-Bevia, Pedro L. Valenzuela, Alejandro Lucia, Alejandro Hernández-Belmonte, Lidia B. Alejo

**Affiliations:** 1 Human Performance and Sports Science Laboratory, Faculty of Sport Sciences, University of Murcia, Murcia, Spain; 2 Department of Sports Sciences. Faculty of Medicine, Health and Sports, Universidad Europea de Madrid, Madrid, Spain; 3 GENUD Toledo Research Group, Faculty of Sports Sciences, University of Castilla-La Mancha. Toledo, Spain; 4 High Performance Sport Center, Region de Murcia, Murcia, Spain; 5 Visma-Bikealease Professional Cycling Team, Den Bosch, Netherlands; 6 Research Institute of Hospital 12 de Octubre (“imas12”), Madrid, Spain; 7 Department of Systems Biology, University of Alcalá, Madrid, Spain

**Keywords:** Strength training, Cycling, Torque, Hypertrophy, Performance, Injury

## Abstract

This randomized controlled trial compared the effectiveness of high-intensity off- and on-bike resistance training (RT) in well-trained cyclists. Thirty-seven cyclists incorporated into their cycling routine a 10-week RT only differing in the exercise used: full squat (off-bike RT, n = 12) or high-intensity all-out pedaling efforts (on-bike RT, n = 12). RT variables like intensity (% maximal dynamic force, MDF), volume, sets, and rest were identical between groups. A third group of cyclists who continued their cycling routine but did not include additional RT stimuli was used as a control (n = 13). The cycling volume at each intensity zone was also matched between the three groups. No significant differences were found between off- and on-bike RT in any parameter. RT groups improved the maximal aerobic power (ES ≥ 0.37) and that attained at the respiratory compensation point (RCP, ES ≥ 0.20). The on-bike RT also significantly enhanced power attained at the ventilatory threshold (ES = 0.24). Off-bike MDF was meaningfully enhanced by both RT groups (ES ≥ 0.16), whereas the on-bike group also significantly increased pedaling MDF (ES = 0.67). Quadriceps size was significantly increased by the off-bike group (ES = 0.22), whereas the on-bike RT also tended to augment this parameter (ES = 0.15) and patellar tendon size (ES = 0.35). Improvements in both RT regimes for time-to-exhaustion capacity (ES ≥ 0.30) were considerable but not significant. The off-bike group tended to increase injury-related symptoms (ES ≥ 0.33). The control group significantly decreased off-and on-bike MDF (ES ≤ -0.40) and quadriceps size (ES = -0.26). These findings suggest that high-intensity on-bike RT is an effective alternative to off-bike RT to safely increase strength, muscle-tendon structure, and cycling performance.

## INTRODUCTION

Resistance training (RT) can increase cycling performance [1], and these improvements are modulated by the volume, the frequency and, particularly, the intensity of the program in question [2]. Indeed, the intensity, commonly expressed as the demand that each load represents relative to an athlete’s maximum muscle strength (e.g., percentage of 1-repetition maximum (1RM)) influences the mechanical and structural muscle tissue adaptations induced by RT. Although similar gains in muscle growth can be obtained with low or high RT loads [3, 4], the latter seem to maximize neuromuscular adaptations and eventual improvements in sports performance [4, 5]. Some cyclists and coaches might be reluctant to incorporate “conventional”, off-bike RT sessions (e.g., squat, deadlift, hip thrust, clean) into training routines. There are indeed concerns on a potential negative effect on actual cycling performance due to increases in muscle mass or fatigue (especially with RT exercises performed to muscle failure [6]) or in risk of lower-back injuries (e.g., induced by using partial ranges of motion [7] or by alterations in spinal curvatures, which are common in these athletes [8]). The need for specific materials outside the usual bicycling setting like bars, discs, or machines can also hinder the implementation of conventional off-bike RT among cyclists, particularly when training away from their habitual facilities. In this effect, the so-called “torque” training (i.e., low-cadence efforts performed against an allegedly high pedaling intensity) has been proposed as an alternative to on-bike RT [9]. Although this approach can be of great practical value (as no additional material is needed) and is highly specific (as it is performed on the cyclist’s own bike), evidence to date regarding its effectiveness in improving neuromuscular and cycling performance is inconsistent [10–12]. The relative force achieved during traditional torque training is overall low (≤ 50% of the cyclists’ maximal pedaling force) [13], which in turn might explain why the desired neuromuscular adaptations might not be attained using this method [10–12]. Additionally, to compare off-bike versus on-bike RT the intensity of both training modalities must be properly equaled [14].

A recent randomized controlled trial (RCT) from our group indicated that on-bike and off-bike RT interventions (using solely high-intensity pedal strokes or full squats, respectively, both at the same relative intensity) induced similar improvements in SQ and pedaling-specific strength compared to a control group performing no RT, as well as in performance in the Wingate test [15]. In the present study, we compared the effects of these two RT interventions on other important variables related to cycling performance, namely injury-related symptoms, endurance capacity (maximal aerobic power, ventilatory thresholds, time to exhaustion, cycling efficiency) and neuromuscular adaptations (patellar tendon thickness).

## MATERIALS AND METHODS

### Experimental design

**FIG. 1:**
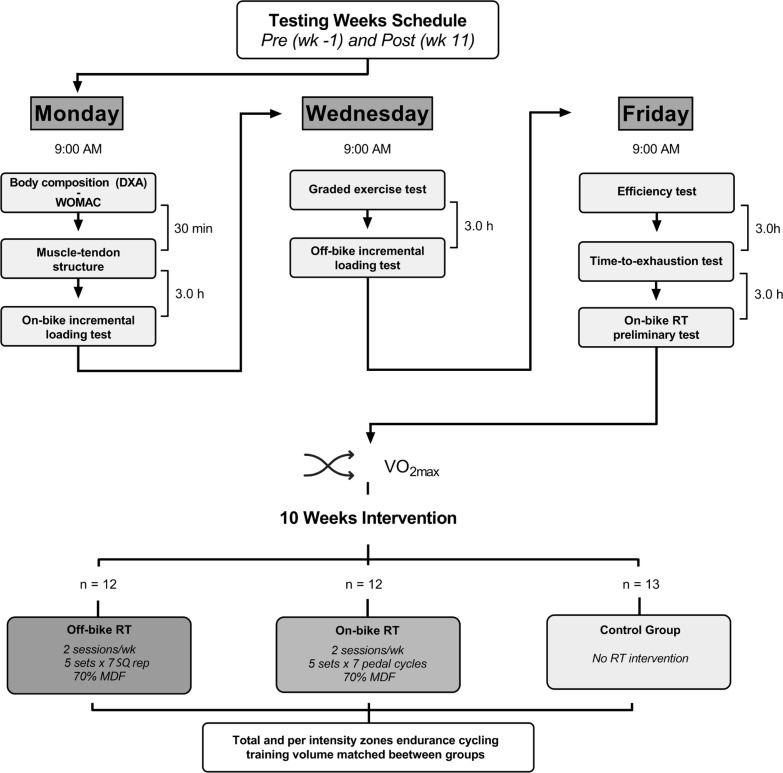
Diagram of the testing sessions and study design. WOMAC, Western Ontario and McMaster Universities; DXA, Dual-energy X-ray absorptiometry. ${\dot{\text{V}}\text{O}}_{2\max}$, Maximal oxygen uptake; MDF, Maximal dynamic force. On-bike resistance training (RT) preliminary test referred to the determination of the chainring-sprocket combination that produced an average of 70% MDF throughout 7 pedaling cycles (only for the on-bike RT group).

This was an ancillary analysis of the aforementioned RCT [15]. The study design is summarized in Figure 1. Participants were randomized to control, off-bike RT (full-squat), or on-bike RT (high-intensity pedaling) group. The intervention lasted 10 weeks in all three groups, who followed the same cycling training program during this period, as detailed below. On the other hand, the loading intensity, volume, number of sets, inter-set and between-session recoveries of the RT sessions were identical in both RT groups as also detailed below. None of the outcomes assessed here were reported in our previous study [15].

### Subjects

Thirty-seven highly trained male cyclists (see [15] for more details) volunteered to participate in the study. Inclusion criteria were: i) having a maximum oxygen uptake (${\dot{\text{V}}\text{O}}_{2\max}$) ≥ 55 ml · kg^−1^ · min^−1^ [16], ii) having at least two years of experience executing the SQ exercise at maximal intended velocity and full range of motion, iii) not taking dietary supplements known to influence physical performance, iv) not having physical limitations, disease, or health problems that could affect the testing or training sessions, and v) not conducting any other resistance exercise during the time this research lasted. Subjects were allocated through stratified randomization into each group according to their ${\dot{\text{V}}\text{O}}_{2\max}$(Figure 1). Descriptive characteristics of study participants are shown in Table 1. Cyclists had a cycling experience of 10.5 ± 5.4 years (5.0 ± 0.7 sessions per week during the last season). The study was approved by the Ethical Committee of the Local University (ID: 3792/2022), and all procedures were conducted following the standards established by the Declaration of Helsinki and its later amendments.

**TABLE 1:** Descriptive characteristics by group

	Off-bike RT	On-bike RT	Control	*P-value*
${\dot{\text{V}}\text{O}}_{2\max}$(ml · kg^−1^ · min^−1^)	62.1 ± 6.4	62.0 ± 7.5	63.0 ± 6.1	0.919
Age (years)	31.8 ± 8.8	32.3 ± 10.8	34.3 ± 10.6	0.823
BMI (kg/m^2^)	22.2 ± 1.6	21.4 ± 1.8	21.9 ± 1.7	0.509
DXA-determined muscle mass (%)	83.4 ± 3.6	81.9 ± 5.3	80.7 ± 3.3	0.270
DXA-determined fat mass (%)	13.7 ± 2.6	14.3 ± 4.6	15.1 ± 3.4	0.601

Abbreviations: ${\dot{\text{V}}\text{O}}_{2\max}$, maximum oxygen uptake; BMI, body mass index; DXA, dual-energy X-ray absorptiometry; RT, resistance training. Off-bike RT, high-intensity squats beside the cycling routine, n = 12; On-bike RT, high-intensity pedal strokes beside the cycling routine, n = 12; Control, removing RT from the cycling routine, n = 13).

### Intervention

In addition to their usual cycling training regime, the off- and on-bike RT groups completed a 10-week RT program with an identical frequency (2 sessions per week), number of sets (5 per session), interset recoveries (4 minutes), between-sessions rest (72 hours), intraset volume (7 repetitions or pedal cycles), and intensity (70% of maximal dynamic force (MDF, see below)), and constant programming model [17]. Therefore, both RT regimes differed solely in the exercise mode used to perform the RT sessions:

– *Off-bike RT group*. Participants performed the squat exercise on a Smith machine. To execute this exercise, they started from an upright position, knees and hips fully extended, and feet placed parallel (shoulder width) on the floor. From this position, they were required to descend the load in a continuous movement until reaching the full squat position. When this point was reached and without pausing between the eccentric and concentric phases, subjects were required to lift the load at a maximal intended velocity until reaching the aforementioned upright position. Both intensity and intra-set volume of the squat exercise were programmed by using the level of effort strategy, which has proven to be a precise, reliable, and practical alternative to velocity-based training for programming RT [18]. Thus, subjects had to put a weight on the bar with which they could lift for a maximum of 15 repetitions (70% MDF, i.e., 1RM), but they only had to complete 7. Completing half of the total repetitions possible against 70% 1RM would result in a velocity loss of ~20% [18]. This level of intraset fatigue has been shown to be an effective and efficient stimulus to promote lower-limb neuromuscular adaptations [19, 20]. All sets were supervised by two experienced researchers who verified adequate compliance with the aforementioned training parameters and gave feedback to the participants when appropriate.
– *On-bike RT group*. Participants in this group performed seated high-intensity pedaling cycles. Each set consisted of 7 maximal voluntary pedaling cycles (i.e., all-out efforts) from a stationary start on a constant 6% slope (~100 m). On an adjustable road test bike (Giant TCR Advanced 1, Taichung, Taiwan) mounted with the Rotor 2INpower power meter [21], researchers determined during a preliminary session (Figure 1 – Friday, Supplemental material 1) the chainring-sprocket combination (e.g., 53 × 13, 53 × 12, or 53 × 11) that produced an average of 70% MDF throughout 7 pedaling cycles. Then, based on the close relationship previously found between force and cadence (r = 0.98) [22], this last parameter was used along the training intervention to program and monitor intensity on their own bike (i.e., similar to velocity-based barbell training). To match the target intensity, cyclists had to modify the gear through the intervention when the target average cadence deviated by ± 5 rpm (~2.5% MDF) due to performance changes. For example, a cyclist had to use 53 × 13 combination during the first 6 weeks to achieve the target cadence of 43 rpm (preliminary determined) and changed to the 53 × 12 one for the last 4 weeks when cadence ≥ 48 rpm.
– Control group: Participants in this group continued their cycling training regime and did not include any RT stimuli during the study.

The cycling training volume in the different intensity zones throughout the study period was matched across the three groups. Intensity zones were determined using power output (PO) ‘zones’ according to participants’ results in the graded exercise test performed at baseline (and described further below): zones I (PO below the ventilatory threshold (VT, see below)), II (PO between the VT and the respiratory compensation point (RCP, see below)), and III (PO above the RCP). Compliance with the target zones was verified by analyzing each participant’s cycling training sessions with the WKO5 software (Peaksware LLC, Lafayette, CO).

### Outcome measures

The following assessments (displayed in the order they were done) were performed in all participants before (baseline – “Pre”) and after the 10-week intervention period (“Post”). Both pre and post-intervention assessments lasted 3 days in total (Figure 1). At both time points, each evaluation was conducted at a similar time of the day (± 1 h) to control the effects of circadian rhythms [23] and under similar environmental conditions (21–22°C and 53–62% humidity).

### Body Composition

Body mass and height were measured using a scale and stadiometer (Seca 784; Hamburg, Germany). Fat and muscle mass levels were assessed using dual-energy X-ray absorptiometry (Hologic QDR series Discovery, Bedford, MA) as described elsewhere [24]. All evaluations were made after at least 8 hours of fasting and with the participants euhydrated.

### Injuries, joint discomfort levels and functional capacity

The Western Ontario and McMaster Universities (WOMAC) questionnaire was used to assess changes in lower-limb discomfort, functionality and injuries. This questionnaire, which has been previously used by us for the same purpose [7, 25], is composed of queries referring to three categories: stiffness, pain, and functional capacity. The sum of all the queries included in each category was considered for further analysis.

### Muscle-tendon structure

The panoramic option of an ultrasound device (Logiq S7, GE Healthcare, Chicago, IL) was used to examine the changes in the anatomical cross-sectional area of the quadriceps femoris (QUAD_CSA_) of the dominant leg. Once at the laboratory, subjects rested supine on an examination bed with their knees fully extended (0° flexion), arms outstretched on both sides of the body, and forearms in a prone position. An experienced sonographer (> 400 hours of experience with this technique, as well as already proven low acquisition and analysis errors [26]) marked and measured the target region corresponding to 50% of the distance between the greater trochanter and mid-patella. The QUAD_CSA_ obtained in this thigh region has been proven to be highly valid and reliable [26]. Reference guides were adhered to the participant’s skin on both sides of the target region to avoid possible deviations during the image acquisition. For each participant, this region was registered on a transparent acetate sheet at baseline to be traced back onto their skin at postintervention. The B-mode option of the same ultrasound device was also used to measure patellar tendon thickness (PT_Thickness_) of the same leg. This parameter was acquired with the subject seated, his knee at 90º (checked by a goniometer), and the probe longitudinal to the tendon length.

The QUAD_CSA_ was obtained by tracing the contour of the whole muscle group (i.e., analyzing in combination the *vastus lateralis, vastus medialis, vastus intermedius*, and *rectus femoris*) using the polygon selection of the software ImageJ (v1.53a, National Institute of Health, USA). The average value resulting from two images was considered for further analysis, measuring a third image when the coefficient of variation of the two averaged values was > 5%. The ImageJ software was also used to measure PT_Thickness_ (distance from the superficial to the deep layers of the tendon). This parameter resulted from the average value of the thickness at three tendon regions: insertion of the inferior layer of the tendon with the patella, 50% of the patella-tibia distance, and insertion of the inferior layer of the tendon with the tibia.

### Off- and on-bike maximal dynamic force

Incremental loading tests were used to measure the off-bike and on-bike MDF. The off-bike MDF was determined through a velocity-monitored incremental test in the squat exercise. The initial load (20 kg) was gradually increased by 10 kg until the mean propulsive velocity was ≤ 0.63 m · s^−1^ (~70% of the MDF, i.e., 1RM). Thereafter, the highest load lifted was used to estimate the MDF as detailed elsewhere [27]. Three repetitions were executed for light (≥ 0.84 m · s^−1^) and two for medium (< 0.84 m · s^−1^) loads. Inter-set rest intervals were 3 min for both load magnitudes. Only the best repetition (the fastest and correctly executed) at each load was considered for subsequent analysis. Participants were required to perform the concentric phase of each repetition at a maximal velocity and the eccentric phase at a controlled velocity (between 0.50–0.70 m · s^−1^), with a 2-s pause between both phases to increase reliability [28]. The velocity was recorded using a validated linear transducer (T-Force System, Ergotech, Murcia, Spain) [29].

The on-bike MDF was measured using an incremental test performed on a friction-loaded cycle ergometer (Monark© 874E, Varberg, Sweden) mounted with a crank power meter (Rotor 2INpower, Madrid, Spain; 50 Hz) [21]. The saddle and handlebar positions of the cycle ergometer were individually adjusted to replicate the cyclist’s own bike. This on-bike incremental loading test has been explained in detail elsewhere [22]. Briefly, the initial load (2 kg) was progressively increased by 0.5–3 kp in each trial through calibrated disks until reaching the heaviest load with which the cyclist could properly perform a whole (360º) pedaling cycle. Load increments were individualized so that participants reached their MDF in less than 8 attempts, interspersed by 5-min rests (i.e., 2-min, free-cadence active recovery against 1 kp followed by 3-min passive recovery). The maximum torque (force in N multiplied by the crank length, 0.175 m) registered during the cycle of the heaviest load was considered as the on-bike MDF.

### Graded Exercise Test

Participants performed a ramp protocol starting at 50 or 100 W depending on their estimated maximal aerobic power (MAP), with subsequent workload increases of 25 W · min^−1^ until volitional exhaustion or when pedaling cadence fell below 60 rpm. Gas exchange data were collected breath-by-breath (MetaLyzer 3B-R3, Cortex Biophysik GmbH; Leipzig, Germany). The VT was determined as the PO at which an increase in both the ventilatory equivalent for oxygen (VE·VO^−1^) and end-tidal partial pressure of carbon dioxide (PetCO_2_) occurred with no concomitant increase in the ventilatory equivalent for carbon dioxide (VE·VCO^−1^) whereas the RCP (also termed ‘second VT’) corresponded to the PO at which both VE·VO^−1^ and VE·VCO^−1^ increased together with a decrease in PetCO_2_ [30]. The MAP was defined as the lowest PO value associated with the ${\dot{\text{V}}\text{O}}_{2\max}$, with the latter considered as the highest VO_2_ value (1-minute mean) attained during the test. This test was performed in a hyperbolic mode (i.e., the work rate was imposed to the subjects with a constant load independently of the pedaling cadence) by attaching each cyclist’s bicycle to the validated Cycleops Hammer ergometer (CycleOps, Madison, WI) [31].

### Efficiency Test

This submaximal test was performed after fasting for at least 8 hours. Cyclists pedaled (seated position-free cadence) for 5 minutes at 50, 60, 70, and 80% of their MAP (as determined in the above-described graded exercise test), respectively. The VO_2_, VCO_2_, and respiratory exchange ratio values between the 3^rd^ and 5^th^ minute of each stage were collected breath-by-breath (MetaLyzer 3B R3, Cortex Biophysik GmbH; Leipzig, Germany) and averaged for further analysis. Delta efficiency was calculated from the slope of the relationship between the work accomplished and the energy expended, as detailed elsewhere [32]. The minimal contribution of anaerobic energy pathways throughout each stage was checked by considering a respiratory exchange ratio value ≤ 1.

### Time-to-Exhaustion Test

The participants conducted a time-to-exhaustion test at the PO corresponding to the RCP. Subjects chose their preferred cadence and were blinded to any variable that could affect their performance (e.g., elapsed time or heart rate). The test started with a 10-minute warmup (5 minutes at 80% and then at 90% of the PO attained at the VT, respectively) and ended when pedaling cadence fell below 60 rpm [33]. The rate of perceived exertion scale (6–20 points) was administered to cyclists at the end of each test to verify maximality. To consider possible changes in RCP-associated PO between baseline and postintervention, the results of this test were expressed as PO*time-to-exhaustion / 1000 (in kJ). All time-to-exhaustion tests were also conducted in a hyperbolic mode by installing the cyclist’s own bike in the Cycleops Hammer ergometer. During the test, air ventilation was controlled with a fan positioned 1.5 m lateral to the subjects at a wind velocity of 2.55 m · s^−1^. At both time points, the same evaluator monitored the test and encouraged the cyclists to perform their maximum effort by using identical feedback.

### Statistical analyses

Normality and homoscedasticity were verified with Shapiro-Wilk and Levene’s tests, respectively. One-way analysis of variance was used to identify the possible differences between the three groups regarding cycling training time in the different intensity zones. A 3 (groups: off-bike RT, on-bike RT, control) × 2 (time: baseline and postintervention) factorial analysis of covariance adjusted by the score of each outcome at baseline was conducted to examine between-group differences. Bonferroni’s post hoc adjustment was used when significant differences (p ≤ 0.05) were detected. The effect size (ES) was computed as Hedges’g and rated as: trivial (0.0–0.19), small (0.20–0.49), moderate (0.50–0.79), or large (≥ 0.80) [34]. The percentage of change (Δ) was calculated as mean of postintervention value minus mean of baseline value / mean baseline value) × 100. Statistical analyses were performed using the SPSS software (version 26.0, IBM Corp, Armonk. NY), and figures were designed using the GraphPad Prism software (version 6.0, GraphPad Software Inc).

## RESULTS

Compliance with the training program was 100% for all the subjects and no dropouts were registered. No significant between-group differences were found when comparing the total cycling volume nor that performed in the different intensity zones throughout the intervention (Table 2).

**TABLE 2:** Cycling training volume per week of the three groups.

	Off-bike RT	On-bike RT	Control	P-value
Total training (hours)	10.7 ± 0.6	10.9 ± 1.4	10.7 ± 1.3	0.876
Training volume < VT (hours)	7.2 ± 0.6	7.7 ± 0.7	7.4 ± 1.7	0.398
Training volume VT-RCP (hours)	2.9 ± 0.8	2.7 ± 1.0	2.8 ± 1.4	0.803
Training volume > RCP (hours)	0.5 ± 0.3	0.6 ± 0.3	0.5 ± 0.2	0.747

RCP, respiratory compensation point; RT, resistance training; VT, ventilatory threshold. Off-bike RT, high-intensity squats beside the cycling routine, n = 12; On-bike RT, high-intensity pedal strokes beside the cycling routine, n = 12; Control, removing RT from the cycling routine, n = 13).

### Injuries, joint discomfort levels and functional capacity

No significant time-by-group interaction or within-group changes were detected for discomfort and functional capacity levels (Table 3). The off-bike group tended to increase pain (ES = 0.33) and stiffness (ES = 0.37), although without reaching significance.

**TABLE 3:** Changes in lower-limb pain, stiffness, and functional capacity by group.

	Off-bike RT				On-bike RT				Control				*P*-value *Time by Group*

	Pre	Post	P-value	ES	Pre	Post	P-value	ES	Pre	Post	P-value	ES	
Pain	5.7 ± 1.8	6.3 ± 1.8	0.208	**0.33**	5.4 ± 0.7	5.3 ± 0.6	0.764	**-0.15**	6 ± 1.9	6.2 ± 2.9	0.526	**0.02**	***0.548***
Stiffness	2.8 ± 1.3	3.3 ± 1.4	0.102	**0.37**	2.6 ± 0.9	2.5 ± 0.8	0.583	**-0.12**	2.5 ± 0.7	2.7 ± 1.5	0.755	**0.15**	***0.298***
Functional capacity	18.5 ± 3.8	20.3 ± 6.3	0.275	**0.36**	17.4 ± 1.1	18.5 ± 2.1	0.259	**0.69**	18.5 ± 3.4	20.5 ± 9.6	0.162	**-0.14**	***0.982***

Abbreviations: ES, effect size; RT, resistance training. Off-bike RT, high-intensity squats beside the cycling routine, n = 12; On-bike RT, high-intensity pedal strokes beside the cycling routine, n = 12; Control, removing RT from the cycling routine, n = 13).

### Muscle-tendon structure and body composition

**FIG. 2:**
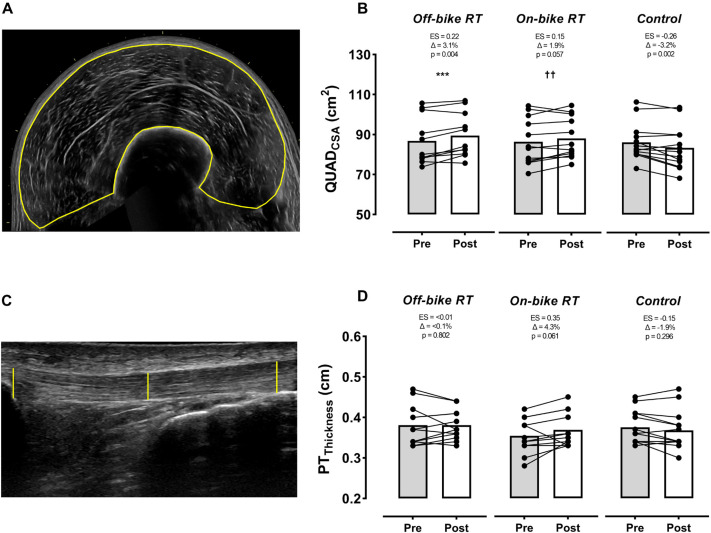
Changes by group in the quadriceps and patellar tendon size. Off-bike RT, high-intensity squats beside the cycling routine, n = 12; On-bike RT, high-intensity pedal strokes beside the cycling routine, n = 12; Control, removing RT from the cycling routine, n = 13). Abbreviations: QUAD_CSA_, quadriceps cross-sectional area; PT_Thickness_, patellar tendon thickness; ES, effect size. Symbols: Δ, percentage of change from pre-training (baseline); * and † indicate significant between groups differences: off-bike RT vs. control group *** (p < 0.001); on-bike RT vs. control group †† (p < 0.01). The p-value below ES and Δ indicates the within-group effect.

Both RT groups increased QUAD_CSA_ (ES ≥ 0.15), but only muscle growth produced by the off-bike group reached significance. A significant time-by-group interaction (p < 0.001 to 0.01) was found for QUAD_CSA_ between RT programs and the control group, which significantly reduced muscle size (p = 0.002, ES = -0.26) (Figure 2). On the contrary, no significant time-by-group interaction or within-group changes were found for PT_thicknes_, although the on-bike group tended to considerably increase this parameter (ES = 0.35).

### Maximal dynamic force

Both RT groups significantly (p ≤ 0.044) improved the off-bike MDF (+8.1 kg and 4.0 kg for the off-bike and on-bike groups, respectively). The on-bike (+12 N, ES = 0.67, p < 0.001), but not the off-bike RT group (+4 N, ES = 0.26), also significantly enhanced the on-bike MDF. A significant time-by-group interaction was found when comparing the two RT groups to the control group (p < 0.001 to 0.01), which significantly decreased both off- and on-bike MDF (p ≤ 0.001, ES ≤ -0.40).

### Graded exercise test

**FIG. 3:**
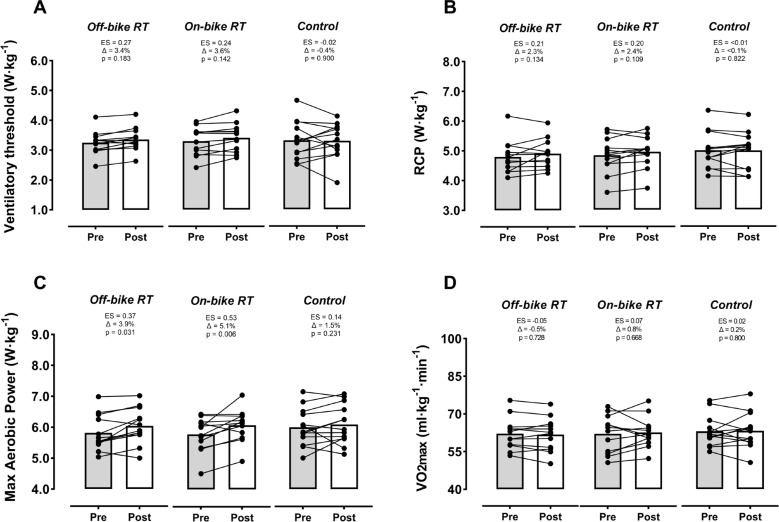
Changes by group in physiological landmarks derived from the graded exercises test. Off-bike RT, high-intensity squats beside the cycling routine, n = 12; On-bike RT, high-intensity pedal strokes beside the cycling routine, n = 12; Control, removing RT from the cycling routine, n = 13). Abbreviations: RCP, respiratory compensation point; ${\dot{\text{V}}\text{O}}_{2\max}$, maximum oxygen uptake; ES, effect size. Symbol: Δ, percentage of change from pre-training (baseline). The p-value below ES and Δ indicates the within-group effect.

No significant time-by-group interaction was found for any of the physiological landmarks assessed throughout the graded exercise test (Figure 3). Nevertheless, both groups incorporating RT exhibited significant benefits for MAP (p ≤ 0.03, ES ≥ 0.37), and a non-significant benefit towards improvement for VT (ES ≤ 0.27) and RCP (ES ≤ 0.21).

### Cycling efficiency and time to exhaustion

**FIG. 4:**
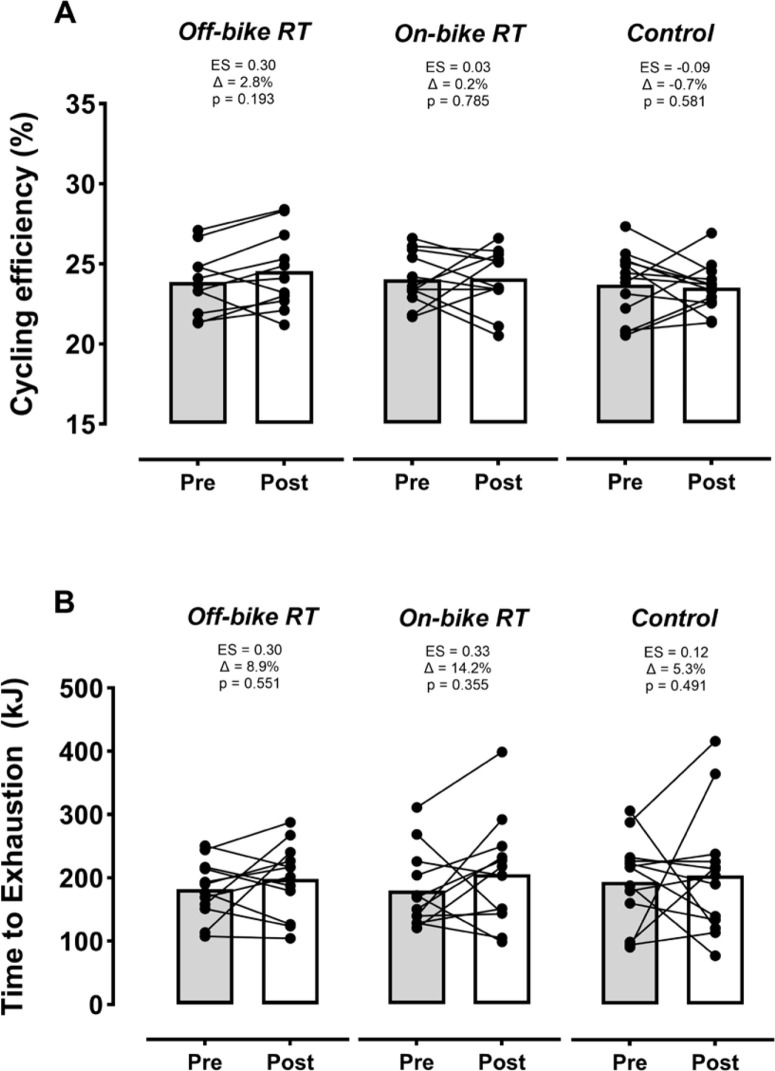
Changes by group in cycling efficiency and time to exhaustion at the respiratory compensation point. Off-bike RT, high-intensity squats beside the cycling routine, n = 12; On-bike RT, high-intensity pedal strokes beside the cycling routine, n = 12; Control, removing RT from the cycling routine, n = 13). Abbreviation: ES, effect size. Symbol: Δ, percentage of change from pre-training (baseline). The p-value below ES and Δ indicates the within-group effect.

No significant time-by-group interactions were found for cycling efficiency and time-to-exhaustion capacity (Figure 4). Within-group changes experienced by RT regimes for time-to-exhaustion capacity (ES ≥ 0.30) and by the off-bike RT for cycling efficiency (ES = 0.30) were considerable but not significant.

## DISCUSSION

The present study indicates that on-bike RT, consisting of high-intensity pedal strokes performed at the maximal intended effort, could be an alternative to traditional off-bike RT for the improvement of strength, muscle-tendon structure, and cycling performance in well-trained athletes. Moreover, we found that removing RT from routines of well-trained cyclists meaningfully declined their lower-limb strength and muscle mass, thus highlighting the need to maintain this type of stimulus (either on or off the bike) throughout the competitive season.

Both RT methodologies (off- and on-bike) assessed in the present study significantly improved the squat 1RM. These increased strength levels could be associated with improvements in cycling performance indicators, especially those dependent, at least partly, on the cyclist’s capacity to apply force at lower cadences (i.e., the RCP and especially the MAP, Figure 3B-C). Nevertheless, no significant improvements were found for the ${\dot{\text{V}}\text{O}}_{2\max}$[35, 36] or cycling efficiency [37, 38] (Figures 4D and 4A). On the other hand, a novelty of our study is that we assessed the effects of RT on delta efficiency (a more accurate indicator than gross efficiency since it removes the metabolic processes not contributing to the actual work [32]). In this regard, our results and those found by other medium-term interventions for gross efficiency [10], suggest that RT programs lasting ≤ 10 weeks would be insufficient to really improve this parameter in well-trained cyclists (probably because their capacity for intermuscular coordination involved during pedaling is already highly optimized [39]). As widely proven after prolonged endurance training periods [40, 41], future studies should examine whether applying our proposed on-bike RT method in the long term could meaningfully improve cycling efficiency. Changes produced by both RT groups in time-to-exhaustion at RCP could be considered meaningful in practice (especially those achieved by the on-bike group, Δ = 14.2%, Figure 4B). However, the high variability of this parameter, also found by previous research [33], could have hindered the achievement of actual statistical significance. The enhancements our study found for this parameter would agree in magnitude and direction with those reported by previous studies for time-to-exhaustion (+17%) [35] or time trials (+6%) [37] at similar intensity domains (i.e., RCP or MAP). For the first time, our results suggested that this performance indicator could also be considerably and safely increased by a RT approach that does not require cyclists to get off the bike (i.e., on-bike RT). Although benefits we observed for most of the performance-related variables (i.e., efficiency, ${\dot{\text{V}}\text{O}}_{2\max}$, PO at the different physiological landmarks) could be relevant in practice, increases in time-to-exhaustion would represent the most evident and direct evidence proving the positive transference of RT (either off or on the bike) on cycling performance (as in any other endurance modality).

Regarding muscle-tendon adaptations, our findings are in line with previous research supporting the effectiveness of high-intensity squat training, even performed far from failure, to increase QUAD_CSA_ [25]. Another novelty of our study stems from the fact that we found that both QUAD_CSA_ (p = 0.057) and PT_thickness_ (p = 0.061) could also be potentially increased when these RT stimuli are performed on the bike (Figure 2). Indeed, these nonsignificant trends for PT_thickness_ increments, together with the no compressive and shearing stress on the spine, could partly explain the fact that the on-bike did not increase the levels of lower-limb discomfort (Table 3). By contrast, the off-bike RT group reported higher (although not significant) lower-limb pain and stiffness values after the intervention program, which is in agreement with previous research using a squat-based intervention [7]. These adverse effects generated when implementing high-intensity (≥ 70% of 1RM) squat training in a population (i.e., cyclists) where alterations in spinal curvatures and low core strength are not uncommon [8, 42] could lead to a further increase in long-term back injuries, which can also be prevalent (~20%) in cyclists [42]. Therefore, although no injuries were recorded throughout the intervention for any group (off-bike RT, on-bike RT, or control), these discomfort-related results suggest that on-bike RT could be used as an alternative to high-intensity squat-based RT to promote gains in the muscle strength, specific performance, and muscle-tendon structure of well-trained cyclists.

Unlike previous investigations implementing “torque training” with low loads (< 50% of 1RM) [13] and submaximal efforts [10–12], our on-bike RT methodology is based on high-intensity pedal strokes with a maximum voluntary effort. Using a high relative intensity (i.e., high % of 1RM) allowed us to analyze the effectiveness of torque training implemented with the most effective loads to maximize strength gains (> 60% 1RM) [4, 5]. In addition, this key variable was accurately programmed and monitored in both groups by a practical application derived from the velocity-based method (i.e., the load-cadence relationship in the case of cycling) [22], thereby exhaustively isolating the main independent variable (RT exercise). Secondly, the proposed an on-bike RT method also required the cyclists to perform the pedal strokes at maximal voluntary effort, thereby differing from most previous torque interventions based on submaximal prolonged efforts (e.g., 4–6 min continuous pedaling) [11, 12]. As reported by previous studies [43, 44], adaptations induced by maximal contractions (i.e., as hard as possible) would be superior to those generated by submaximal ones, probably due to enhanced neural changes.

Secondary results also showed significant reductions of both off- and on-bike maximal dynamic force, as well as a QUAD_CSA_ atrophy, when well-trained cyclists stop their RT, even if they continue their cycling training regime (Table 2). Although these negative effects did not translate into significant losses for the examined cycling parameters, they could potentially affect other specific indicators mainly related to anaerobic capacity (e.g., mean and power PO during a Wingate test) [15, 45]. Therefore, our results suggest that well-trained cyclists should maintain off- or on-bike RT throughout the season to minimize any eventual performance decline.

This study was not exempt from limitations. Firstly, it is unknown whether applying these RT stimuli over a period longer than 10 weeks, using other programming models (e.g., linear or undulated) or degrees of effort (e.g., very low or very high), could alter our findings. Secondly, we decided to program a fixed number of repetitions to be completed by both training groups to equal their RT volume. Like the velocity loss approach for off-bike RT, it would be necessary to extend the knowledge on possible methods to program and control intra-set volume during on-bike RT for increasing the accuracy of fatigue prescription during off- and on-bike comparisons. Finally, although including a control group made up of resistance-trained cyclists allowed researchers to inform about the negative effects of ceasing the RT stimulus, future studies are encouraged to complement this analysis by including a control group with no RT experience.

## CONCLUSIONS

Our findings indicate that on-bike RT based on high-intensity pedal strokes performed at a maximal intended effort could be an alternative to traditional off-bike RT to safely increase strength, muscle-tendon structure, and specific performance in well-trained cyclists.

## SUPPLEMENTAL MATERIAL 1.

Video illustrating the preliminary on-bike test to identify the gear that produced an average of ~70% MDF throughout 7 pedaling cycles (53 × 12 for the cyclist shown in the video). Link: https://www.youtube.com/watch?v=FLHRLIfriTI
